# Predictors of Sexual Victimization Among Autistic and Non-Autistic College Students

**DOI:** 10.1007/s10803-023-06064-w

**Published:** 2023-07-22

**Authors:** Natalie Libster, Connie Kasari, Alexandra Sturm

**Affiliations:** 1 Department of Education, University of California Los Angeles, Los Angeles, USA; 2 Semel Institute for Neuroscience, University of California Los Angeles, Los Angeles, USA; 3 Department of Psychological Science, Loyola Marymount University, Los Angeles, USA

**Keywords:** Autism, Higher education, ADHD, Unwanted sexual contact, Sexual assault

## Abstract

Purpose. This study examined predictors of sexual victimization among autistic and non-autistic college students. Specifically, we aimed to determine whether autistic students are more likely than non-autistic students to experience unwanted sexual contact and sexual assault, controlling for co-occurring diagnoses. We also aimed to determine whether students with other disabilities, specifically ADHD, learning disability (LD), and psychological disorders, are more likely than students without these disabilities to experience unwanted sexual contact and sexual assault. Methods. Autistic students (n=270) and non-autistic students (n=270) who had participated in a nationwide survey were matched on demographic characteristics and co-occurring diagnoses. Logistic regression analyses were conducted to address the research questions and evaluate predictors of unwanted sexual contact and sexual assault. Results. Autistic students were as likely as non-autistic students to have experienced unwanted sexual contact and sexual assault, controlling for co-occurring diagnoses. Regardless of autism diagnostic status, students with ADHD were more likely than students without ADHD to have experienced unwanted sexual contact and sexual assault. Conclusions. Although autism diagnostic status was not a significant predictor of unwanted sexual contact or sexual assault, other factors associated with increased risk of sexual victimization, such as co-occurring ADHD, are likely to be found in autistic populations. This study highlights the importance of educational, social, and institutional supports to decrease sexual victimization among college students with neurodevelopmental disabilities.

## Introduction

The structural inequalities that people with disabilities often experience may put them at-risk for victimization (Roulstone et al., 2011; Forster & Pearson, 2020; Pearson et al., 2022a). People with disabilities regularly experience discrimination, social exclusion, and oppression that result from societal ideals of normalcy and the social privilege of able-bodiedness (Davis, 2016; O’grady et al., 2004). This imbalance of power has been shown to affect the relationships of autistic adults, who experience heightened rates of interpersonal victimization (Griffiths et al., 2019; Papadopoulos, 2016). For example, an autistic participant in a study by Pearson et al. (2022a) reported, “Because I could not support myself, I had to live with (and please) men who were willing to have a live-in girlfriend. This trapped me in relationships where I had none of the power” (p. 506). Autistic adults have further expressed difficulty recognizing their intuition or “gut feeling” about safety in relationships, and they have emphasized the importance of learning how to identify non-verbal danger cues and how to establish boundaries in relationships (Fardella et al., 2018). In addition, autistic adults have reported that the desire to please others has led to interpersonal victimization. An autistic participant in a study by Pearson et al. (b) stated, “(I) have been pressured into doing things that I wasn’t comfortable with to try to please others who I thought were friends” (p. 146). This abuse of power may also characterize the relationships of autistic college students, putting them at increased risk for sexual victimization.

Sexual victimization is a critical and prevalent issue across college campuses. Among college students in the U.S., 26% of women and 7% of men have experienced nonconsensual sexual contact by force or inability to consent (Cantor et al., 2020). Students with certain disabilities are also more likely than students without disabilities to experience sexual victimization on campus (Cantor et al., 2020). The number of college students on the autism spectrum continues to increase (Buescher et al., 2014), yet it is unclear whether these students are at increased risk for sexual victimization. A systematic review by Dike et al. (2022) found that 22 studies examining sexual victimization among autistic youth and adults have yielded inconsistent results. Nine of the reviewed studies found higher rates of sexual victimization among autistic groups compared to non-autistic groups, whereas the remaining studies did not find significant differences in sexual victimization between autistic and non-autistic groups (Dike et al., 2022). Two studies that have examined sexual victimization among autistic college students have also yielded inconsistent results. Brown et al. (2017) found that autistic students and non-autistic students with disabilities reported higher rates of sexual victimization compared to students without disabilities, while Rothman et al. (2021) found similar rates of sexual victimization among autistic and non-autistic students. Methodological inconsistencies across these studies, including differences in sample size, participant characteristics (e.g., gender identity), and self-report measures, may have contributed to the discrepant results.

The mixed findings of these studies may have also been attributed to the presence of other disabilities in the autistic and non-autistic groups. In their study, Brown et al. (2017) compared two disability groups – one of autistic students and one of non-autistic students with other disabilities – and a control group of students without any disabilities. The two disability groups reported similar levels of sexual victimization, and both groups reported higher rates of sexual victimization than the control group. Meanwhile, Rothman et al. (2021) did not control for the presence of other disabilities in the non-autistic group and identified similar rates of sexual victimization between autistic and non-autistic students. The inconsistent results found by Brown et al. (2017) and Rothman et al. (2021) highlight the importance of controlling for other disabilities when examining sexual victimization among autistic and non-autistic college students. Autistic and non-autistic students who have certain disabilities may be at increased risk of sexual victimization. For example, adults with ADHD experience higher rates of intimate partner violence (Guendelman et al., 2016; Wymbs et al., 2019) and sexual victimization (Snyder, 2015; Wymbs & Gidycz, 2021) compared to adults without ADHD. Adults with ADHD therefore experience similar imbalances of power that often characterize the relationships of autistic adults (Snyder, 2015; Waite, 2010).

The first aim of the current study was to examine whether autistic college students are more likely than non-autistic students to experience unwanted sexual contact and sexual assault, controlling for co-occurring diagnoses. As many as 70–79% of autistic adults meet criteria for at least one co-occurring psychiatric condition (Buck et al., 2014; Kentrou et al., 2021; Lever & Geurts, 2016; Moss et al., 2015; Rosen et al., 2018). Anxiety, depression, and ADHD are among the most prevalent co-occurring psychiatric conditions in autistic adults, with an estimated prevalence of 27–42% for any anxiety disorder, 23–37% for depression, and 20–37% for ADHD (Hollingdale et al., 2020; Hollocks et al., 2019; Lai et al., 2019). Since certain psychiatric conditions, such as ADHD, are associated with increased risk of sexual victimization (Snyder, 2015; Wymbs & Gidycz, 2021), it was important to control for these disabilities in the autistic and non-autistic groups. The second aim of the study was to examine whether students with other disabilities, specifically ADHD, learning disability (LD), and psychological disorders, are more likely than students without these disabilities to experience unwanted sexual contact and sexual assault. Since prior research has shown that undergraduate women who belong to a greater number of clubs and organizations experience higher rates of sexual victimization (Mustaine & Tewksbury, 2002), it was also important to control for level of social participation when analyzing predictors of unwanted sexual contact and sexual assault.

## Methods

### Participants

The current study analyzed secondary data collected by the Cooperative Institutional Research Program (CIRP) Diverse Learning Environments (DLE) survey, which was developed and maintained by the Higher Education Research Institute (HERI) at the University of California, Los Angeles. The DLE survey is administered annually to undergraduate students who have acquired 24 credit hours at 2-year colleges as well as second- and third-year students at 4-year institutions. The survey is used to evaluate campus climate, assess student learning outcomes, and explore institutional practices as experienced with faculty and staff (Higher Education Research Institute, 2022).

Beginning in 2017, the DLE survey asked students, “Do you have any of the following disabilities or medical conditions?” Possible responses included *learning disability (dyslexia, etc.)*, *attention-deficit hyperactivity disorder (ADHD)*, *autism spectrum disorder*, *physical disability (speech, sight, mobility, hearing, etc.)*, *chronic illness (cancer, diabetes, autoimmune disorders, etc.)*, *psychological disorder (depression, anxiety, PTSD, etc.)*, or *other*. Students selected all applicable responses. The current sample was drawn from 53,166 students who completed the DLE between 2017 and 2020. Of these students, 473 reported having a diagnosis of autism. Participants in the current study (N = 540) were selected using the case control fuzzy procedure in SPSS version 25. Students who reported a diagnosis of autism (n = 270) were matched to a control group that did not report a diagnosis of autism. Matching criteria included gender (man and woman), race/ethnicity (White and students of color), sexual orientation (heterosexual and non-heterosexual), family income (< 30k, 30k-50k, 50k-100k, and > 100k), and college/university.

Students were matched on binary dimensions of gender, sexual orientation, and race/ethnicity due to the limited number of students who belonged to gender, sexual, and racial/ethnic minority groups. In the 2017 and 2018 surveys, response options for gender identity included *man, woman, trans man, trans woman, genderqueer/gender-nonconforming*, and *different identity (free response)*. In the 2019 and 2020 surveys, response options for gender identity included *man/trans man, woman/trans woman, genderqueer/gender-nonconforming*, and *different identity (free response).* For the purposes of this study, students who selected *woman* or *trans woman* (2017–2018) and *woman/trans woman* (2019–2020) were coded in the *woman* gender category, and students who selected *man* or *trans man* (2017–2018) and *man/trans man* (2019–2020) were coded in the *man* gender category. Students who selected *genderqueer/gender-nonconforming* and *different identity* were excluded from the study due to the limited number of responses.

Furthermore, in the 2017–2020 surveys, response options for sexual orientation included *heterosexual/straight, gay, lesbian, bisexual, queer*, and *other (free response).* The 2018–2020 surveys also included *asexual* and *pansexual* as response options. For the purposes of this study, students who selected *heterosexual/straight* were coded in the *heterosexual* category, and students who selected the other responses options were coded in the *non-heterosexual* category. Finally, in the 2017–2020 surveys, response options for race/ethnicity included *American Indian or Alaskan Native, East Asian (e.g., Chinese, Japanese, Korean, Taiwanese), Filipina/o/x, Southeast Asian (e.g., Cambodian, Vietnamese, Hmong), South Asian (e.g., Indian, Pakistani, Nepalese, Sri Lankan), Other Asian, African American/Black, African, Caribbean, Other Black, Native Hawaiian or Other Pacific Islander, Mexican American/Chicana/o/x, Puerto Rican, Central American, South American, Other Hispanic or Latina/o/x, Middle Eastern, European*, and *Other White.* The 2020 survey also included *other (please specify)* as a response option. For the purposes of this study, students who selected *European* and *Other White* were coded in the *White* category, and students who selected the other response options were coded in the *students of color* category.

Types of institutions included public and private universities, 4-year colleges, and 2-year colleges. A total of 57 colleges/universities were represented in the sample, and students were matched on the specific institution that they attended. Colleges included technical schools and institutions that offered undergraduate degree programs, whereas universities included institutions that offered undergraduate and graduate degree programs. Autistic and non-autistic students were further matched on reported diagnoses of learning disability (LD), ADHD, and psychological disorders. Autistic students with more than one disability (e.g., ADHD and LD) were matched with non-autistic students with those disabilities. The current study used deidentified data and was certified as exempt from IRB review.

### Measures

#### Demographics

Demographic characteristics included (1) gender (man and woman), (2) race/ethnicity (Non-Hispanic White and students of color), (3) sexual orientation (heterosexual and non-heterosexual), (4) family income (< 30k, 30k-50k, 50k-100k, and > 100k), (5) type of institution (4-year university, 4-year college, and 2-year college), and (6) the presence or absence of each of the following diagnoses: ADHD, LD, and psychological disorders.

#### Social Participation

Students were asked whether they had done any of the following since entering college: *(1) Joined a social fraternity or sorority, (2) Joined an ethnic or culturally-based fraternity or sorority, (3) Joined a racial/ethnic student organization, (4) Played intercollegiate athletics (e.g., NCAA or NAIA-sponsored), (5) Joined a club or organization related to your major, (6) Joined an LGBTQ + student organization*, or *(7) Joined a student-run political club*. Response options included *yes* and *no*. To account for potential conflation between ethnic/culturally-based fraternities and sororities and racial/ethnic student organizations, these two activities were combined into a single social group. If students responded *yes* to *Joined an ethnic or culturally-based fraternity or sorority* or *Joined a racial/ethnic student organization*, responses were coded as *yes* to joining a racial/ethnic student group. In addition, students were asked how often they participated in study groups since entering college. Response options included *frequently, occasionally*, and *not at all.* For the purposes of this study, responses were dichotomized into *yes* (*frequently* or *occasionally)* and *no (not at all)*. Social participation scores were then calculated for each student. Social participation scores, which ranged from 0 to 7, were the total number of social groups/organizations to which students belonged: social fraternity or sorority, racial/ethnic student group, intercollegiate athletics, major-related organization, LGBTQ + organization, political club, or study group.

#### Sexual Contact And Sexual Assault

Students were given the following prompt:

“The following questions ask about unwanted sexual contact and sexual assault. Please keep the following definitions in mind when answering the questions:

#### Unwanted Sexual Contact

*Non-verbal behavior*: *unwanted exposure to pornography, unwanted filming or taking photographs of a sexual nature, unwanted videos/photos of a sexual nature, sexual gestures made at you, unwelcome sexual advances*

*Verbal behavior*: *sexual comments, sexual rumors, threats to commit sexual acts, threatening to use physical force*

*Physical contact*: *brief intentional touching, brief grabbing of a sexual nature, brief physically intimidating behavior*

#### Sexual Assault

*Committed or attempted acts of a sexual nature or sexual intercourse occurring without the victim freely giving consent or against someone who is unable to consent or refuse. The acts can include (but are not limited to)*:

- *Forced touching (e.g., kissing, groping, fondling) or being forced to touch the perpetrator or someone else*.
- *Sexual intercourse or attempted sexual intercourse – vaginal, anal, or oral penetration or attempted penetration of the victim or victim being forced to penetrate the perpetrator or someone else*.

Any of the above occurring due to intimidation or misuse of authority where the victim was pressured to consent.”

To measure unwanted sexual contact, students were asked, “Since you entered college, have you had any unwanted sexual contact?” Response options included *yes* and *no*. To measure sexual assault, students were asked, “Since you entered this college, has someone sexually assaulted or attempted to sexually assault you?” Response options included *yes* and *no*.

### Analysis

#### Descriptive Characteristics

Descriptive statistics and bivariate tests were conducted to examine the success of the case-control matching procedure. The autistic and non-autistic groups were compared on each of the matched demographic variables using chi squared tests for categorical variables (i.e., gender, race/ethnicity, sexual orientation, income, type of institution, and the presence of ADHD, LD, and psychological disorders).

#### Preliminary Analyses

Two separate logistic regression analyses were conducted to determine whether demographic variables predicted unwanted sexual contact and sexual assault. Basic assumptions for logistic regression, such as the absence of multicollinearity and influential outliers, were met when selecting the demographic predictors. The presence of a disability (autism, ADHD, LD, or a psychological disorder) and sexual orientation were not included in the final models to meet these assumptions. Of the students in the sample who did not have a disability, only one had experienced unwanted sexual contact and one had experienced sexual assault. Furthermore, a Fisher’s exact test revealed a significant association between gender and sexual orientation (p < .001). The majority of men in the sample (80%) were heterosexual, while the majority of women (54%) were non-heterosexual.

Predictors of unwanted sexual contact and sexual assault included gender, race/ethnicity, income, and type of institution (see Table 1). The regression models revealed that gender was a significant predictor of unwanted sexual contact and sexual assault. Women were 6.82 times more likely than men to have experienced unwanted sexual contact (p < .001) and were 9.94 times more likely than men to have experienced sexual assault (p < .001). 24% (n = 65) of women compared to 5% (n = 13) of men had experienced unwanted sexual contact, and 15% percent (n = 40) of women compared to 2% (n = 5) of men had experienced sexual assault. Race/ethnicity, income, and type of institution were not significant predictors of unwanted sexual contact or sexual assault.

**Table 1:** Demographic Predictors of Unwanted Sexual Contact and Sexual Assault

Outcome		Predictor	OR	Lower OR	Upper OR	*p*
Unwanted Sexual Contact						
		Gender*	6.82	3.69	13.52	< 0.001
		Race	1.00	0.55	1.78	0.99
		Family income				
		Under 30k	-	-	-	-
		30k-50k	0.37	0.12	0.95	0.06
		50k-100k	1.06	0.55	2.07	0.86
		Over 100k	1.35	0.66	2.78	0.41
		Type of Institution				
		4-Year University	-	-	-	-
		4-Year College	1.22	0.69	2.22	0.51
		2-Year College	0.83	0.32	2.03	0.70
Sexual Assault						
		Gender*	9.94	4.12	29.64	< 0.001
		Race	1.07	0.50	2.21	0.85
		Family income				
		Under 30k	-	-	-	-
		30k-50k	0.27	0.04	1.04	0.10
		50k-100k	1.16	0.51	2.65	0.72
		Over 100k	1.33	0.54	3.24	0.53
		Type of Institution				
		4-Year University	-	-	-	-
		4-Year College	1.73	0.83	3.90	0.16
		2-Year College	0.83	0.21	2.83	0.78

#### Unwanted Sexual Contact and Sexual Assault

Two separate logistic regression analyses were then conducted to examine predictors of unwanted sexual contact and sexual assault, controlling for total social participation. Predictors of unwanted sexual contact and sexual assault included autism diagnostic status, ADHD diagnostic status, LD diagnostic status, psychological disorder diagnostic status, and social participation score. Since gender was found to be a significant predictor of unwanted sexual contact and sexual assault in the preliminary analyses, gender was included as a covariate in the final models. Both models were conducted using R version 4.1.0.

## Results

### Descriptive Characteristics

Descriptive characteristics of the sample are reported in Table 2. The majority of autistic and non-autistic students were Non-Hispanic White (67%), heterosexual (63%), attended a 4-year college (55%) and had a psychological disorder (59%). The sample was approximately 50% men and women.

**Table 2:** Demographic Characteristics of Autistic and Non-Autistic Students

Variable		Autistic		Non-Autistic	*p*
*N*		270		270	
Gender: Men, *N* (%)		136 (50%)		136 (50%)	0.99
Race: Non-Hispanic White, *N* (%)		182 (67%)		182 (67%)	0.99
Sexual Orientation: Heterosexual, *N* (%)		170 (63%)		170 (63%)	0.99
Family Income					
Under 30k, *N* (%)		73 (27%)		71 (26%)	0.85
30k-50k, *N* (%)		37 (14%)		44 (16%)	0.40
50k-100k, *N* (%)		80 (30%)		84 (31%)	0.71
Over 100k, *N* (%)		80 (30%)		71 (26%)	0.39
Type of Institution					
4-Year University		76 (28%)		76 (28%)	0.99
4-Year College		149 (55%)		149 (55%)	0.99
2-Year College		45 (17%)		45 (17%)	0.99
Co-occurring diagnosis					
Learning disability, *N* (%)		57 (21%)		57 (21%)	0.99
ADHD, *N* (%)		79 (29%)		79 (29%)	0.99
Psychological disorder, *N* (%)		158 (59%)		158 (59%)	0.99

### Unwanted Sexual Contact and Sexual Assault

14% (n = 78) of the total sample, including all diagnostic and non-diagnostic groups, had experienced at least one form of unwanted sexual contact. Results of the regression analysis revealed that ADHD diagnostic status – but not autism diagnostic status – was a significant predictor of unwanted sexual contact (see Table 3). Students with ADHD were 1.98 times more likely than those without ADHD to have experienced unwanted sexual contact (95% CI [1.09, 3.56], p = .02). 22% (n = 34) of students with ADHD compared to 12% (n = 44) of students without ADHD had experienced unwanted sexual contact. Meanwhile, 8% (n = 45) of the total sample had experienced sexual assault. Results of the logistic regression analysis revealed that ADHD diagnostic status – but not autism diagnostic status – was a significant predictor of sexual assault (see Table 3). Students with ADHD were 2.16 times more likely than those without ADHD to have experienced sexual assault (95% CI [1.02, 4.54], p = .04). 13% (n = 21) of students with ADHD compared to 6% (n = 24) of students without ADHD had experienced sexual assault. McFadden’s pseudo-R-squared value was 0.22 for the model predicting unwanted sexual contact and 0.25 for the model predicting sexual assault, indicating good model fit (McFadden, 1974).

**Table 3:** Predictors of Unwanted Sexual Contact and Sexual Assault

Outcome	Predictor	OR	Lower OR	Upper OR	*p*
Unwanted Sexual Contact					
	Autism	1.07	0.62	1.86	0.80
	ADHD*	1.98	1.09	3.56	0.02
	LD	1.35	0.72	2.50	0.34
	Psychological disorder	1.66	0.87	3.31	0.13
	Social participation score*	1.51	1.26	1.83	< 0.001
	Gender*	5.75	2.98	12.00	< 0.001
Sexual Assault					
	Autism	0.58	0.28	1.16	0.13
	ADHD*	2.16	1.02	4.54	0.04
	LD	0.99	0.44	2.16	0.99
	Psychological disorder	2.56	1.06	7.23	0.05
	Social participation score*	1.60	1.28	2.00	< 0.001
	Gender*	7.37	2.96	22.51	< 0.001

### Co-occurring ADHD Diagnostic Status

Since ADHD was found to be a significant predictor of unwanted sexual contact and sexual assault, two additional logistic regression models were conducted to examine whether the likelihood of experiencing sexual victimization differed among students with ADHD only and students with a combination of ADHD and other diagnoses. Students’ co-occurring ADHD diagnostic status was coded based on whether they identified as having (1) ADHD only, (2) ADHD and ASD, (3) ADHD and LD, (4) ADHD, ASD, and LD, or (5) no ADHD. ADHD only was coded as the reference group in order to compare sexual victimization among students who had ADHD with and without co-occurring diagnoses. Since the directionality of the relationship between psychological disorder and sexual victimization is unclear, this variable was included as a covariate in the models. Predictors of unwanted sexual contact and sexual assault included co-occurring ADHD diagnostic status, gender, psychological disorder diagnostic status, and social participation score. To determine whether women were more likely than men to experience sexual victimization as a function of co-occurring ADHD diagnostic status, an interaction between gender and co-occurring ADHD diagnostic status was included in the models.

The interaction between gender and co-occurring ADHD diagnostic status was not a significant predictor in either model – this interaction variable was therefore taken out of the final models to facilitate the interpretability of the results. The results of the final models are depicted in Table 4. Students without ADHD were 0.40 times less likely than students with ADHD only to have experienced unwanted sexual contact (95% CI [0.17, 1.00], p = .04) and were 0.32 times less likely than students with ADHD only to have experienced sexual assault (95% CI [0.12, 1.00], p = .04). Students who had ADHD and one or more co-occurring diagnosis were as likely as students with ADHD only to have experienced sexual victimization. McFadden’s pseudo-R-squared value was 0.22 for the model predicting unwanted sexual contact and 0.25 for the model predicting sexual assault, indicating good model fit (McFadden, 1974).

**Table 4:** Co-occurring ADHD Diagnostic Status as a Predictor of Unwanted Sexual Contact and Sexual Assault

Outcome	Predictor	OR	Lower OR	Upper OR	*p*
Unwanted Sexual Contact					
	Gender*	5.92	3.07	12.31	< 0.001
	Psychological disorder	1.70	0.89	3.39	0.11
	Social participation score*	1.53	1.28	1.86	< 0.001
	Co-occurring ADHD status				
	ADHD only	-	-	-	-
	ADHD + ASD	0.72	0.22	2.32	0.58
	ADHD + LD	0.96	0.29	3.16	0.95
	ADHD + ASD + LD	0.80	0.23	2.75	0.73
	No ADHD*	0.40	0.17	1.00	0.04
Sexual Assault					
	Gender*	7.34	2.97	22.29	< 0.001
	Psychological disorder*	2.60	1.07	7.37	0.04
	Social participation score*	1.62	1.30	2.04	< 0.001
	Co-occurring ADHD status				
	ADHD only	-	-	-	-
	ADHD + ASD	0.64	0.14	2.69	0.55
	ADHD + LD	1.02	0.26	4.04	0.97
	ADHD + ASD + LD	0.32	0.06	1.50	0.16
	No ADHD*	0.32	0.12	1.00	0.04

## Discussion

The first aim of the current study was to examine whether autistic college students were more likely than non-autistic students to experience unwanted sexual contact and sexual assault, controlling for co-occurring diagnoses. We found that when controlling for co-occurring diagnoses, autistic students were not more likely than non-autistic students to experience unwanted sexual contact or sexual assault. The second aim of the study was to examine whether students with other disabilities, specifically ADHD, LD, and psychological disorders, were more likely than students without these disabilities to experience unwanted sexual contact and sexual assault. Regardless of autism diagnostic status, students with ADHD were more likely to have experienced unwanted sexual contact and sexual assault than students without ADHD. Our secondary models revealed that students who had ADHD and one or more co-occurring diagnosis were as likely as students with ADHD only to have experienced sexual victimization. These findings support prior research demonstrating that undergraduate students with ADHD experience higher rates of sexual victimization than students without ADHD (Snyder, 2015; Wymbs & Gidycz, 2021).

Autistic and non-autistic students in the current study reported similar levels of unwanted sexual contact (n = 44 vs. n = 34, respectively) and sexual assault (n = 21 vs. n = 24, respectively), controlling for co-occurring diagnoses. As previously discussed, autistic adults often have co-occurring diagnoses, such as ADHD, that are associated with increased risk of sexual victimization (Hollingdale et al., 2020; Lai et al., 2019; Wymbs and Gidycz, 2021). This may explain the inconsistent findings of the current study and those of Brown et al. (2017). Brown et al. (2017) found higher rates of sexual victimization among autistic students compared to students without disabilities, but the researchers did not control for co-occurring diagnoses that may have increased autistic students’ risk of sexual victimization. While Rothman et al. (2021) found similar rates of sexual victimization between autistic and non-autistic students, the researchers did not control for co-occurring diagnoses in the autistic group or for the presence of other disabilities in the non-autistic group. The two groups therefore may have had similar rates of psychiatric conditions that are associated with increased risk of sexual victimization. Autistic and non-autistic students with disabilities have been shown to report similar levels of sexual victimization (Brown et al., 2017). The current study found that ADHD, in particular, is associated with increased risk of sexual victimization among autistic and non-autistic college students.

Although increased risk of unwanted sexual contact and sexual assault was not observed among autistic students in the present sample, autistic adults may have difficulty identifying abusive behaviors from romantic and sexual partners (Fardella et al., 2018; Sedgewick et al., 2019). In a study by Sedgewick et al. (2019), several autistic women explained that they tended to assume “the best of people” (p. 118) and had experienced domestic abuse or sexual victimization repeatedly because “there’s that whole ulterior motive thing that I end up missing” (p. 118). An autistic participant in a study by Fardella et al. (2018) also reported, “If I am bullied, or taken advantage of… the person gets to do it for a while because it takes me a long time to realize what they are doing… it takes me a while to make the connection that they are behaving in a way that somewhere in my brain is saying to me is ‘unsafe’” (p. 1467). Autistic students who experience sexual victimization may therefore underreport these occurrences if they are unable to detect abusive behaviors in relationships. It is also important to note increased risk of other sources of interpersonal trauma experienced by autistic college students. Specifically, autistic college students report higher rates of other forms of interpersonal victimization, including emotional and physical victimization, compared to their non-autistic peers (Rothman et al., 2021).

The current study highlights the importance of individual-level and contextual factors that may reduce sexual victimization among college students with disabilities. As previously discussed, students with disabilities often experience structural inequalities in their interpersonal relationships (Roulstone et al., 2011; Forster & Pearson, 2020; Pearson et al., 2022a, b). Autistic adults have expressed the importance of having relationships with people they can trust and turn to for support and protection (Fardella et al., 2018). Prior research has also found that faculty support (Kim et al., 2022) and sense of belonging to the campus community (Rothman et al., 2021) are protective factors against interpersonal victimization among autistic and non-autistic college students. However, college faculty often have limited knowledge of neurodevelopmental disabilities and lack confidence in their abilities to accommodate the needs of students with disabilities (Sniatecki et al., 2015; Zeedyk et al., 2019). Autistic students also report a lower sense of belonging in college compared to non-autistic students (Rothman et al., 2021). These findings highlight the need for greater awareness and acceptance of neurodiversity on college campuses, faculty training on ways to support the needs of students with disabilities, and campus climates that focus on the prevention of sexual violence (Strout et al., 2014).

Students with disabilities may further benefit from resources and supports that teach them how to identify safe versus unsafe behaviors from sexual partners (Fardella et al., 2018). In a study by Fardella et al. (2018), an autistic participant reported, “When teaching about sexual violence you could say ‘Keep an eye out for this, these are risk factors, these are things you want to avoid’. These things may be obvious to a neurotypical person but might not be obvious to someone with ASD” (p. 1468). Knowledge of sexual health has also been shown to be negatively associated with sexual victimization among autistic adults (Brown-Lavoie et al., 2014). Autistic adults demonstrate lower levels of sexual knowledge than non-autistic adults (Brown-Lavoie et al., 2014), and adolescents and adults with ADHD tend to engage in riskier sexual behaviors than those without ADHD, including earlier initiation of sexual activity and more sexual partners (Donahue et al., 2013; Flory et al., 2006; Rokeach & Wiener, 2018). Education on sexual health and safe sex practices, including consent, may therefore further benefit students with disabilities given the structural inequalities that perpetuate sexual violence. Finally, institutional prevention programs and supports for survivors of sexual violence should be tailored to the specific needs of students with disabilities (Bonomi et al., 2018). Among survivors of intimate partner violence, those with disabilities are more likely than those without disabilities to experience internal and external depression, stress, and self-harm behaviors (Scherer et al., 2013, 2016). Prior research further indicates that autistic adults may be at heightened risk of developing post-traumatic stress disorder (PTSD) after experiencing trauma (Haruvi-Lamden et al., 2018; 2020; Rumball et al., 2020). Targeted supports for college students who experience sexual violence should therefore account for the differential impact of interpersonal trauma on students with disabilities.

### Limitations and Future Directions

This study has many strengths, including a relatively large, well-matched sample of autistic and non-autistic college students. It also has limitations, which we hope will be addressed in future research. First, students in the current study were only matched on binary dimensions of gender (man and woman) due to the limited number of gender-diverse students who participated in the 2017–2020 DLE surveys. Future research should account for gender minority status when examining sexual victimization among autistic college students. Not only are gender-diverse individuals at-risk of experiencing sexual victimization (Cantor et al., 2020), but a higher prevalence of gender-diverse individuals are autistic compared to the general population (Warrier et al., 2020). Students in the current study were also matched on binary dimensions of race (Non-Hispanic White and students of color) and sexual orientation (heterosexual and non-heterosexual) in order to maintain a large enough sample. By doing so, the current study failed to capture differences in reported sexual victimization across various racial/ethnic groups and sexual identities, which have been reported in prior studies (Cantor et al., 2020). The generalizability of the current study is further limited by race, as the majority of the participants were White.

The current study implemented a specific case-control matching procedure in which autistic students and non-autistic students were matched on co-occurring diagnosis. As a result, the numbers of autistic and non-autistic students with each co-occurring diagnosis (ADHD, LD, and psychological disorder) and with each combination of co-occurring diagnoses (e.g., ADHD and LD) were the same. The results of the current study may therefore not generalize to a non-controlled college campus environment. Furthermore, the current study compared reported sexual victimization between students *with* a disability (e.g., autism) and students *without* that disability (e.g., no autism). Differences in sexual victimization between students without any disability and students in different diagnostic groups (ASD only, ASD and ADHD, ASD and LD, etc.) were not examined in the current study and are areas for future research.

Another limitation was the inability to include sexual orientation as a predictor in our preliminary models due to the significant association between sexual orientation and gender. The majority of men in the sample were heterosexual while the majority of women were non-heterosexual. Prior research has found that autistic adults are more likely than non-autistic adults to identify as non-heterosexual (George & Stokes, 2018; Gilmour et al., 2012; Pecora et al., 2020a), and that autistic women have higher rates of non-heterosexual orientations than autistic men (Dewinter et al., 2017; Pecora et al., 2020b). This may explain why there were more non-heterosexual women in our sample than non-heterosexual men, since non-autistic students were matched to autistic students on gender and sexual orientation. Since non-heterosexual orientations are associated with increased rates of sexual victimization (Cantor et al., 2020), future research should address whether higher rates of non-heterosexual orientations among autistic women put them at increased risk of sexual victimization. |.

Finally, gender and social participation, which were included as covariates in the current study, were significant predictors of unwanted sexual contact and sexual assault. Psychological disorder, which was a covariate in the secondary models, was also a significant predictor of sexual assault. As previously discussed, the directionality of the relationship between psychological disorder and sexual victimization is unclear – while some studies propose that psychological conditions, such as depression, increase risk for victimization (Krahé & Berger, 2017; Orcutt et al., 2005), other studies have shown that survivors of sexual violence often experience negative mental health outcomes (Campbell et al., 2009; Kilpatrick & Acierno, 2003). Therefore, the effects of gender, social participation, and psychological disorder on sexual victimization among autistic and non-autistic college students are areas of future research.

## Conclusion

The current study demonstrated that students with ADHD, regardless of autism diagnostic status, were more likely to have experienced unwanted sexual contact and sexual assault. Given this finding, future research should aim to understand the institutional-level factors, as well as the individual-level traits of perpetrators, that place students with ADHD at increased risk of sexual victimization. Autistic students in the current study were as likely as non-autistic students to experience unwanted sexual contact and sexual assault, controlling for co-occurring diagnoses. However, other factors associated with increased risk of sexual victimization, such as co-occurring ADHD, are often found in autistic populations. This study therefore highlights the importance of educational, social, and institutional supports to decrease sexual victimization among college students with neurodevelopmental disabilities.

## Data Availability

The dataset analyzed in the current study is not publicly available.
